# Bibliometric analysis of youth myocardial infarction research (1980–2023)

**DOI:** 10.3389/fcvm.2024.1478158

**Published:** 2024-11-26

**Authors:** Yang Fu, Qi Han, Fei Wang, Xiuyun Dong

**Affiliations:** ^1^ Department of Cardiology, Shanxi Cardiovascular Hospital, Taiyuan, China; ^2^ Department of Cardiology, Cardiovascular Disease Hospital of Shanxi Medical University, Taiyuan, China; ^3^ Shanxi Key Laboratory of Otorhinolaryngology Head and Neck Cancer, First Hospital of Shanxi Medical University, Taiyuan, China

**Keywords:** myocardial infarction, youth, clinical treatment, bibliometrics, research frontiers

## Abstract

**Introduction:**

Cardiovascular diseases include myocardial infarction, a high mortality disease. Myocardial infarction patients are becoming younger, typically defined as patients under 45 years of age. This study analyzes the relevant papers on myocardial infarction in youth in the Web of Science Core Collection (WoSCC) between 1980 and 2023.

**Methods:**

It uses bibliometric methods to systematically understand the current status and development trend of research in this field. We searched the WoSCC between 1980 and 2023 for research papers and reviews on myocardial infarction in youth. We set the screening criteria for language as English and used tools such as Citespace, SCImago Graphica, and VOS Viewer to analyze the selected literature exhaustively. This comprehensive approach helped us gain a comprehensive understanding of research hotspots, academic partnerships, and trends in the field.

**Results:**

From the WoSCC, we identified 790 publications related to myocardial infarction in youth. First, the United States, Italy, and China are major contributors to international cooperation. The United States plays a vital bridging role. Next, in the scholars' combined contribution power analysis, Krumholz and Donfrio were the key contributors in this field. In addition, popular research directions are based on age. As a result of the literature cluster analysis, we found that myocardial infarction in youth is associated with gender, smoking, coagulation factors, apolipoproteins, and gene polymorphisms.

**Conclusion:**

This is the first comprehensive bibliometric study of myocardial infarction in youth. It aims to examine the current status and trends in myocardial infarction in youth. As a result, the study results will provide researchers with an overview of emerging trends.

## 1 Introduction

Myocardial infarction is often considered a disease that occurs primarily in the elderly population. However, studies in recent years have shown a gradual increase in myocardial infarction prevalence in the young population (1, 2). The age range of myocardial infarction in the young is currently under considerable debate. Some studies have defined the onset of myocardial infarction in young people as <40 years of age. Others have defined the term “young” as patients under 45 years of age, and individuals considered “very young” as an age group under 35 years of age (3, 4). In recent decades, myocardial infarction has steadily increased in young people due to changes in lifestyle (5, 6), environmental factors (7), and genetic factors (8). Factors such as dietary structure, exercise habits, and stress levels may significantly impact cardiovascular health (9, 10). According to a study, patients with myocardial infarction younger than 50 years of age benefit significantly from lifestyle interventions (11). In addition, trauma of early onset has been recognized as an independent risk factor in individuals with a history of myocardial infarction. It may result in lifelong systemic inflammation (12). External factors such as environmental pollution, chemical exposure, and drug abuse may also increase the risk of myocardial infarction in youth (13, 14). For example, there was a report of an acute myocardial infarction due to stenosis and contracture of blood vessels in a 28-year-old man who had taken amphetamines (15). Last but not least, myocardial infarction clinical manifestations in young people may be atypical and easily overlooked or misdiagnosed (16, 17). This further increases its difficulty and risk. Therefore, an in-depth understanding of the pathogenesis, influencing factors, and clinical features of myocardial infarction in young people is essential for the timely prevention and treatment of myocardial infarction in young people.

Bibliometric analysis is a systematic method to reveal the development dynamics and hot issues in the research field through statistics and analysis of a large amount of literature (18, 19). In the research field of myocardial infarction in youth, bibliometric analysis is of great significance and value. First of all, bibliometric analysis can help us understand the research hotspots, main research directions and international cooperation in this field. Second, the statistical analysis of the number of documents, author collaboration, inter-institutional collaboration, and keyword frequency provides insights into the academic landscape, collaborative network structure, and research hotspots in the study of myocardial infarction in youth. Besides, the authors’ H-index, M-index, and G-index, as well as the number of publications, are used to measure scholarly impact and achievement (20–22). Finally, the bibliometric analysis based on WoSCC database has high credibility and representativeness, covers rich literature resources and research fields, and can provide us with comprehensive and reliable research data and information (23).

This paper explores the current status and trends in youth myocardial infarction research. It does this by conducting a bibliometric analysis of relevant literature in the WoSCC database from 1980 to 2024. Overall, in this study, we will comprehensively explore the current status and challenges of youth myocardial infarction research through bibliometric methods. This will be done from the following six perspectives.

(1) We will shed light on the dynamics of youth myocardial infarction research and assess research activities by examining annual publication and citation trends.
(2) We will evaluate their contributions by analyzing the collaborative network of countries, institutions, and authors in the area of juvenile myocardial infarction research.
(3) We will conduct a comprehensive analysis of keywords and co-cited literature related to myocardial infarction in adolescents to identify key themes, research hotspots, and trends.
(4) We identified the top journals in the field by assessing the impact of journals focusing on juvenile myocardial infarction.
(5) We provide an in-depth discussion of the current state of basic and clinical research on myocardial infarction in adolescents.

## 2 Methods

### 2.1 Retrieval strategy

**Figure 1:**
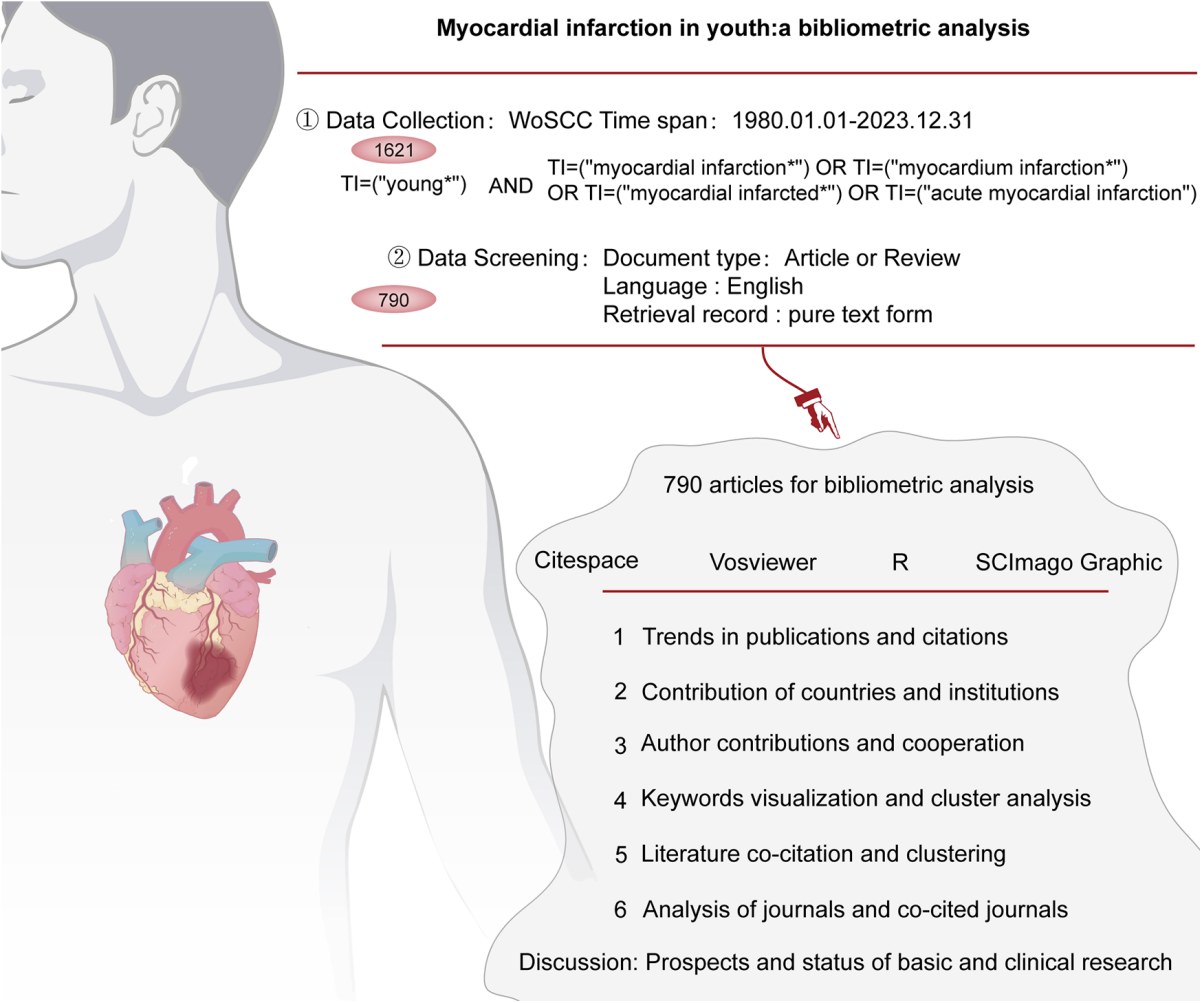
Literature search and data analysis flowchart.

The Web of Science Core Collection (WoSCC) is a comprehensive academic database covering more than 190 subject areas worldwide. WoSCC provides unparalleled literature retrieval and citation analysis services for all disciplines through its accreditation as a top database of peer-reviewed, high-quality scholarly journals covering more than 250 disciplines, including more than 21,100 peer-reviewed, high-quality scholarly journals (18). Therefore, in this study, we used the WoSCC as a data source and searched and downloaded it through the Science Citation Index. Based on the search methodology shown in Figure 1, we searched using the following search terms: TI = “young*” and TI = (“myocardial infarction*”) or (“myocardium infarction*”) or (“myocardial infarcted*”) or (“acute myocardial infarction”). In addition, we limited the year of publication to January 1980 to December 2023, the type of literature to thesis or review, the language to English, and excluded duplicate articles.

### 2.2 Bibliometric terms

Latent Semantic Indexing (LSI) and Log-Likelihood Ratio (LLR) are both critical algorithms in cluster analysis. LSI achieves dimensionality reduction by applying Singular Value Decomposition (SVD) to LSI achieves dimensionality reduction by applying SVD, which reduces the number of features and represents documents by capturing semantic correlations in a low-dimensional space (24). In contrast, LLR analyzes the lexical distributions in the documents through the log-likelihood ratio test to identify clusters of significantly related documents (25).

### 2.3 Bibliometric analysis and visualization

After exporting the retrieved article data, we used bibliometric and visualization analysis tools, including the “BiblioMetrix” package of R software (26), CiteSpace (27), VOSviewer (28), and SCImago Graphic.

#### 2.3.1 Citespace advanced (6.2.R4) and MapEquation

First, we remove duplicates through the “Remove Duplicates” option in CiteSpace. Then, CiteSpace was employed to examine the co-occurrence and cluster analysis of researchers, keywords, and literature co-citations within the domain of myocardial infarction in young adults. First, in the author collaboration analysis, G-index (K = 25) was selected to construct the collaboration network. The clustering analysis was performed by combining the LLR and LSI algorithms with the “All in one” option. Secondly, in keyword analysis, the filtering conditions TOPN = 30, 40, and 50 were selected to build the keyword co-occurrence network and perform thematic clustering analysis. In conclusion, the G-index (K = 25) was employed as the screening criterion to construct the literature co-citation network and analyze thematic clustering analysis, as well as to visualize citation burst strength.

In addition, we exported literature citation data (net format) for the period 1980–2023 via CiteSpace and imported it into the online tool MapEquation to analyze it. We filtered the core literature consistently cited for more than 5 years and generated flow maps. Finally, we reveal the interdisciplinary knowledge flows and citation relationships in the field by overlaying the dual maps. This helps identify research hotspots and multidisciplinary impact pathways.

#### 2.3.2 VOSviewer and SCImago graphic

Statistical analyses of country publication and inter-agency cooperation were conducted using VOSviewer. First, in the analysis of country cooperation, the screening condition was set as the minimum number of literature for a single organization was not less than 12, and 3 clusters were obtained with 19 organizations. Second, in the analysis of institutional cooperation, the screening condition was set as the minimum number of literature for a single organization was not less than 7, and 2 clusters were obtained from a total of 16 organizations. Finally, the above data were imported into SCImago Graphic software to visualize international inter-institutional cooperation.

#### 2.3.3 R4.3.0

We used the “BiblioMetrix” R package to statistically analyze annual publication trends, standardized citation trends, and measure the contributions of the top 10 authors in terms of publications, G-index, M-index, and H-index in the field. In addition, we performed a Bradford's Law analysis of myocardial infarction research in young patients using the bibliometrix R package. As a result, core impact journals in the field were identified.

We first uploaded the zip file by compressing the 790 documents we obtained into a zip file. We then ran “bibliometrix::biblioshiny ()” to open the web tool in R. Finally, a CSV file containing information about authors, institutions, countries, and keywords was exported. Then, we present the frequency and cumulative frequency heatmap visualization of the keywords.

## 3 Results

### 3.1 Trends analysis of the annual number of publications and citations

This study covered January 1980 to December 2023, and 790 documents were screened. The types of literature were dissertations and review papers, written in English, covering 323 journals, collaborated by 3,962 authors, and cited 15,699 references. Moreover, Supplementary Figure S1A illustrates trends in myocardial infarction in young adults. The trend graph of annual publications shows an overall increase with an average growth rate of 3.42% over the past 40 years. It can roughly be categorized into three phases: the first phase (1980–2000) shows a slow growth period, indicating that scholars have become interested in the field of myocardial infarction in the young and are actively exploring the field; the second phase (2000–2020) shows a stable growth period, indicating that the field is still receiving sustained attention from scholars; and the third phase (2020–2023) is an explosive growth period revealing that the subject is highly valued by a wide range of scholars. The subject has received a high degree of attention and enthusiasm from a wide range of scholars, gradually becoming a research hotspot. Overall, myocardial infarction in young people is booming and contributes to modern medical research.

Furthermore, the trend of annual citation frequency in the field of myocardial infarction in young adults is presented in Supplementary Figure S1B, which can be roughly divided into four phases: the first phase (1980–1998) is a fluctuating period; the second phase (1998–2008) is a stabilizing period; the third phase (2008–2017) is a period of explosive growth; and the fourth phase (2017–2023) is a period of slow decline. Combined with the publication trend, we find that the annual citation frequency shows overall growth before 2017, and gradually decreases after 2017. There are several reasons for this phenomenon: (1) Decline in research quality: despite the increase in journals and publishing platforms, research quality is mixed, and low-quality research is rapidly increasing. (2) Fragmentation of research fields: research fields are constantly subdivided, and research content is gradually “fragmented and decentralized”, which fails to attract researchers and thus affects the citation rate. (3) Citation lag: Since it takes time for newly created research content to produce the corresponding effect, it may not be fully cited early, and the citation rate may drop quickly. (4) Changes in citation habits and standards: With the advancement of science and the diversification of information access channels, researchers are no longer limited to traditional journals and pay more attention to high-quality research content, which also leads to a decline in citations.

To summarize the above, the trend of annual publications in the field of myocardial infarction in young people is generally increasing. Particularly after 2020, the field is experiencing rapid growth, indicating rapid development. However, the annual citation trend passes through four phases: a fluctuating period, a stabilizing period, an explosive growth period, and a slow decline period. We have summarized the potential factors that may influence this trend. This will enable us to gain a deeper understanding of the overall development of myocardial infarction in young adults.

### 3.2 Analysis of the contribution of countries and institutions

To assess the research contributions of different countries and institutions in the field of myocardial infarction in the young and to gain insight into the underlying collaborative relationships between them, we used software such as VOSviewer and SCImago Graphic to perform a detailed analysis of the country and institution's publications as well as the countries’ collaborative networks.

In Supplementary Figure S2A, the size of the nodes is proportional to the number of articles published by the country, and a larger node indicates that the country has published more articles in the field. Statistics show the United States, Italy, and China are the top three countries in terms of published articles. The United States published 198 articles, Italy 68 articles, and China 66 articles. The width of the lines connecting the nodes represents the degree of cooperation between countries, with thicker lines indicating closer cooperation between countries (29). The country collaboration network is divided into three clusters, with close collaboration between countries in the same cluster. At the same time, the connecting lines between different clusters indicate the existence of collaborative relationships with each other, with thicker connecting lines indicating stronger cooperation. Therefore, the United States plays a key connecting bridge in these three clusters. Within the same cluster, the U.S. has strong collaborative relationships with the Netherlands and India. Within different clusters, the U.S. works relatively closely with Spain, Canada, the U.K., Italy, India, and the Netherlands. China's collaboration with other countries needs to be strengthened.

Moreover, institutions can be divided into two clusters, with close collaboration within the same cluster (Supplementary Figure S2B). In terms of the size of each node, Yale University, St. Luke's Hospital (USA), Missouri State University, and Harvard University have significant influence in terms of contribution. The connection strength shows that the research collaboration between St. Luke's Hospital USA Yale University and Missouri State University is close. While Yale and Harvard cooperate, Harvard is relatively less involved in the collaborative model. Compton University of Madrid, while contributing relatively little, has relatively close research collaborations with St. Luke's Hospital, Yale University, and Missouri State University. Regardless of the contributions, it is expected that more countries or institutions will propose collaborations and actively participate in the collaborative model in the future. This will advance the field of myocardial infarction in young people globally.

### 3.3 Analysis of author contributions and collaborative network relationships

To assess the extent to which scholars contribute to the field of science, researchers have developed several output metrics, the most widely used of which is the H-index, which takes into account the number of publications made by authors as well as the impact and citations of their papers, and therefore provides a comprehensive measure of their contribution to the field of science (30, 31).

In other words, two scholars with the same H-index may produce similar contributions to science, independently of their number of publications. First, using the “BiblioMetrix” package of R software, the Top 10 were ranked according to the H-index in descending order (Supplementary Table S1). In addition, we analyze author impact by combining three key metrics, Total Citation (TC), Number of Papers (NP) and Publication Year stratification (PY_start). Among them, Krumholz, Harlan M has the highest H-index, suggesting that he has made the most significant contributions to preventing myocardial infarction in young people. The M-index and G-index complement the H-index in assessing scholarly impact, emphasizing citation frequency and paper quality, respectively, but each has field-specific limitations. For example, the H-index is easily affected by the number of papers published, while the M-index and G-index are easily affected by the field of publication. This means that when using these indicators, it is recommended to combine them with other indicators to obtain a comprehensive picture (32). In Supplementary Tables S2–S4, M-index, G-index, and number of published articles by the top ten authors are counted.

**Figure 2:**
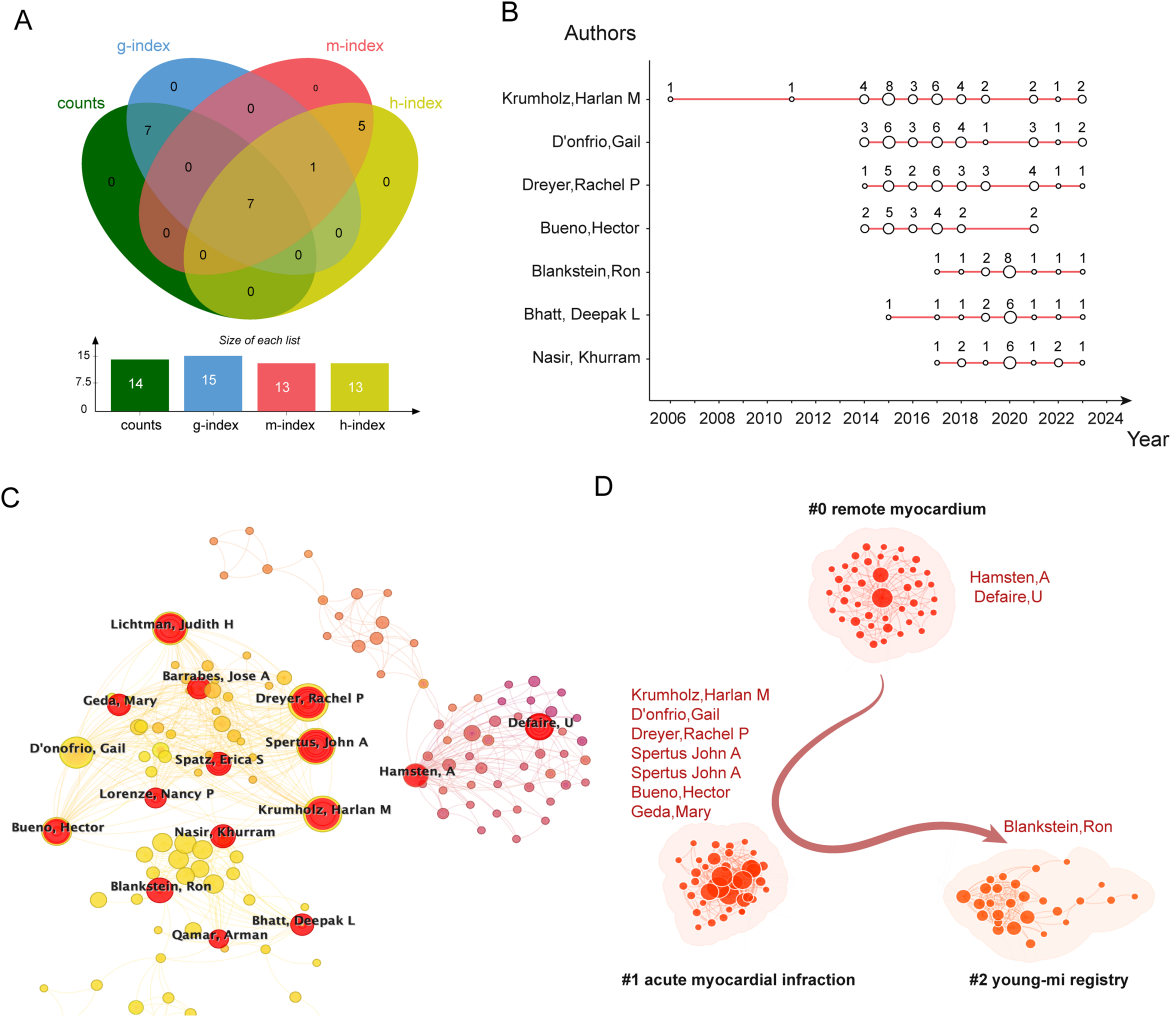
Visual analysis of authors’ contributions. **(A)** Venn diagram analysis of publication volumes, H-index, G-index, and M-index of the TOP 10 authors to identify the 7 scholars with high combined contribution. **(B)** Annual publications of the 7 scholars with high combined contribution. Vertical coordinates of the diagrams denote the number of articles and the total number of publications per year. The horizontal coordinates indicate the number of articles per year. The size of the dots reflects the publications of each scholar. **(C)** Visualization of the top 10 authors in terms of publications with the CiteSpace. **(D)** Cluster analysis of the top 10 authors in terms of publications again with the CiteSpace, which identifies the three clusters representing their research areas.

In Figure 2A, combining the above indexes and obtaining intersections using Venn diagrams, seven scholars were identified, including Krumholz, Harlan, D'onfrio, Gail, Dreyer, Rachel P, Bueno, Hector, Blankstein, Ron, Bhatt, Deepak L, Nasir Khurram, et al. These scholars have demonstrated a large combined academic contribution to the field of myocardial infarction in the young. They had a profound impact on the field's development. Figure 2B illustrates the annual publications of these seven scholars. The top 10 authors in terms of publications were again visualized with CiteSpace software, as shown in Figure 2C. Among the top 10 authors in terms of publications, Krumholz, and Harlan M dominate the list with 31 publications. Donfrio, Gail, and Hamsten, A rank second and third with 27 and 26 publications respectively. Among the authors highlighted in Supplementary Figure S3, Hamsten, A, Defaire, U, and Schwarta, SM are among the top three in terms of article citations. Krumholz, Harlan M has the highest citation burst intensity of 12.06, from 2014 to 2019.

To gain a deeper understanding of the authors’ research interests in myocardial infarction in young adults, we used CiteSpace software to perform LLR cluster analysis and visualization. The results showed that the top 10 authors in terms of publications were clustered in “#0 remote myocardium”, “#1 acute myocardial infarction” and “#2 young-mi registry” , as shown in Figure 2D. Therefore, these three clusters represent the main research directions in the field of myocardial infarction in young adults by the top 10 authors in terms of the number of publications.

### 3.4 Keywords visualization and cluster analysis

**Figure 3:**
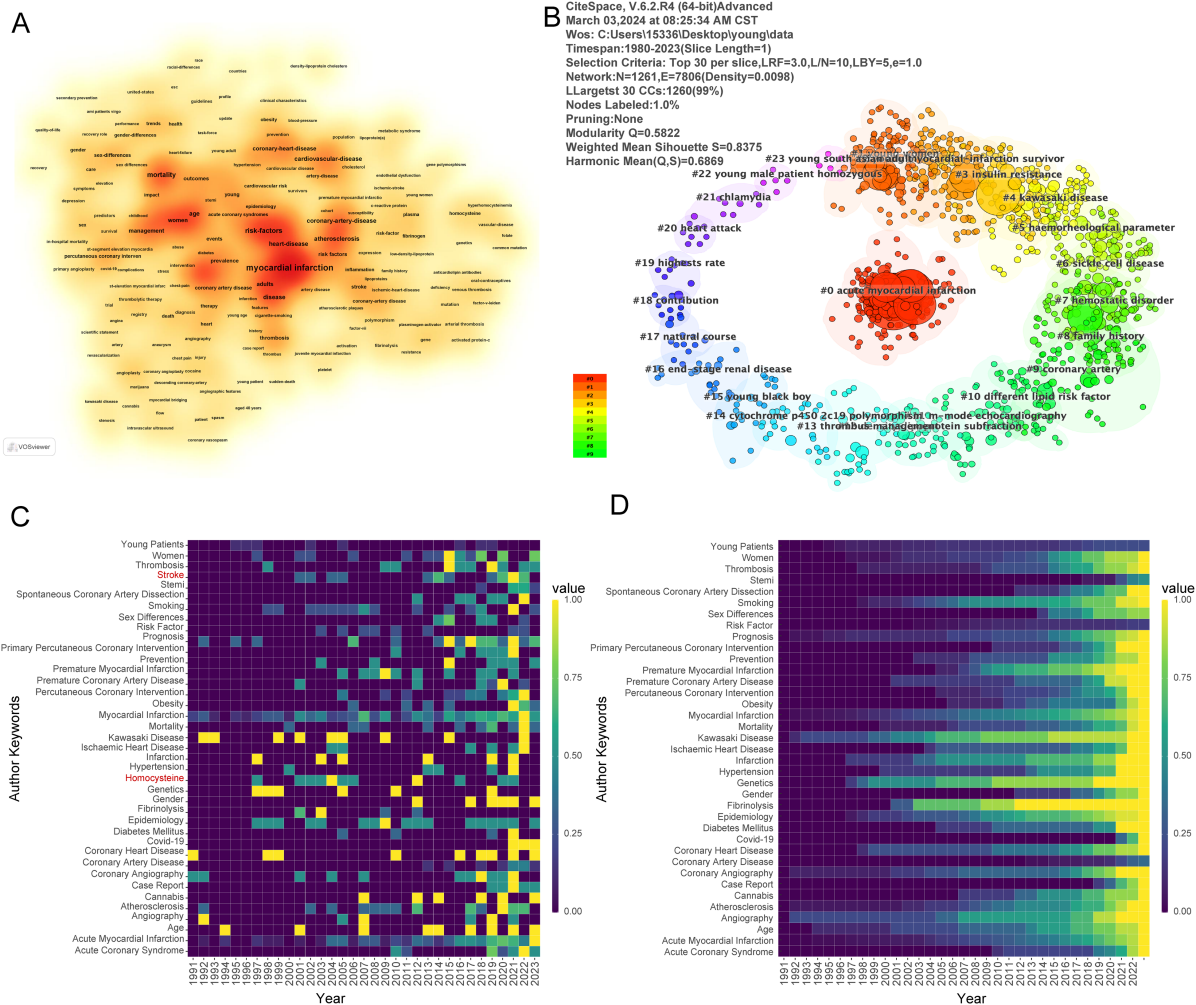
Keyword visualization and cluster analysis. **(A)** VOSviewer generates a keyword heat map, with darker areas indicating higher frequency of occurrence. **(B)** Keyword visualization was performed using CiteSpace software and LLR clustering analysis was used, with the clustering parameter set to TOP *N* = 30, and 23 clusters were obtained. **(C)** Presents the annual keyword frequency, and **(D)** shows the annual keyword cumulative frequency. Although the keyword frequencies have been standardized, the vertical axis represents the keywords and the horizontal axis represents the years. The keywords Stroke, Homocysteine were added to the C graph compared to the D graph.

A keyword co-occurrence analysis of the literature on myocardial infarction in young adults was performed to provide a comprehensive understanding of the research focus in this area. In this study, VOSviewer was first used to generate a keyword heat map (Figure 3A), with darker colors indicating a higher frequency of occurrence, suggesting that the field is receiving widespread attention and development. Keywords such as acute myocardial infarction, coronary heart disease, gender, age, and risk factors have higher frequency and are being widely explored. In addition, we categorized and frequency counted the keywords (Table 1). In the group of risk factors associated with myocardial infarction in youth, coronary heart disease, age, and atherosclerosis were the top three. In the diagnosis and treatment of myocardial infarction in the youth group, percutaneous coronary intervention, angioplasty, and coronary angiography were the most commonly used keywords. In the Research Topics group, C-reactive protein, gene, and activated protein C were the three most popular keywords. Among the risk factors for myocardial infarction in youth, smoking, metabolic syndrome, emotional frustration, and depression were also popular topics for research. These reflect the hotspots and frontiers of development in the field of myocardial infarction in youth.

**Table 1:** Top 10 risk factors, treatments and diagnostics and frequency of keywords for research topics.

Rank	Risk factors	Counts	Treatment and diagnosis	Counts	Study themes	Counts
1	Coronary artery disease	131	Percutaneous coronary intervention	32	C-reactive protein	13
2	Age	71	Angioplasty	13	Gene	12
3	Atherosclerosis	59	Coronary angiography	7	activated protein C	11
4	Sex differences	41	Thrombolytic therary	5	Fibrinogen	8
5	Smoking	14	Cardiovascular medicine	4	Coagulation factor v	8
6	Cholesterol	7	Cardiovascular prevetion	2	inflammation	5
7	Depression	7	Coronary artery surgery	2	Apolopoprotein b	5
8	Metabolic syndrome	6	Intravascular ultrasond	2	Factor v leiden	3
9	Cocaine	5	Drug therapy	1	Apoptosis	2
10	Diabetes mellitus	5	Bypass surgery	1	Oxidative stress	2

Moreover, CiteSpace software was again used for keyword visualization and analysis. LLR clustering analysis was used, setting the clustering parameter as TOP *N* = 30/40/50. 23, 22, and 22 clusters were obtained based on sorting out synonyms and eliminating extraneous terms (Figure 3B, Supplementary Figures S4, S5, Supplementary Tables S5–S7), respectively. The visualization of TOP *N* = 30 results showed that the top three clusters were “#0 acute myocardial infarction”, “#1 young women”, and “#2 young myocardial infarction survivors” based on the analysis of the number of keywords; in the TOP *N* = 40 visualization results, the top three clusters were “#0 ST-segment elevation”, “#1 young women”, and “#2 young subjects”; while in the TOP *N* = 50 visualization results, the top three clusters were “#0 sex different”, “#1 young survivors”, and “#2 haemorheological parameter”. This suggests that gender and age are still the hotspots and trends of research in the field of myocardial infarction in youth.

Finally, we used the visual analysis method of the annual keyword frequency heatmap. We explored research trends and cutting-edge hotspots in myocardial infarction in young adults using a fuzzy set theory topic network. Figures 3C,D illustrate the normalized frequencies of author keywords as annual keyword frequencies and cumulative annual keyword frequencies, respectively. The annual keyword frequency has increased by two keywords, Stroke and Homocysteine, over the annual keyword accumulation frequency. In the group of risk factors associated with myocardial infarction in young adults, keywords such as age, gender, diabetes, obesity, and coronary heart disease highlighted the popular directions of research. Notably, COVID-19 has also emerged as an emerging research direction of increasing interest due to the novel coronavirus pandemic in 2020.

In the research topics group, terms such as fibrinogen and genetics reflect major trends in research. In the diagnostic and therapeutic group, keywords such as coronary angiography and percutaneous coronary intervention highlight the main diagnostic and therapeutic tools. In Supplementary Figure S6, the upper right-hand edge presents the “Motor Themes keywords”, which include “thrombophilia”, “c-reactive protein”, “c-reactive protein”, and “c-reactive protein”, “interleukin-6”, “lipoprotein(a)” and other keywords. These keywords have significant centrality and high density in the research topic of myocardial infarction in youth, which signifies that they play an important role in the research direction of the field, and are the frontier and hotspot of the research topic of myocardial infarction in youth. The upper left limit represents the “Niche Themes Keywords”, including the keywords “young and middle-aged”, and “cholesterol”. The upper left limit indicates “Niche Themes Keywords”, including the keywords “young and middle-aged”, “cholesterol”, “lipoproteins” and so on, which are linked to the relatively cold research direction in the field of myocardial infarction in young people. The lower right limit represents the “Basic themes keywords”, which illustrate the basic direction of myocardial infarction research topics in young people. The lower left limit represents “Emerging or Declining themes keywords”, which are relatively loosely connected with other keywords and belong to emerging research directions in the field of myocardial infarction in youth. Myocardial infarction in youth clustering and visualization analysis provides a comprehensive overview of research hotspots.

### 3.5 Literature co-citation and cluster analysis

Through clustering analysis of the literature co-citations, we have deeply analyzed the structure of the relationship between the research hotspots and the related subfields, providing solid theoretical support for revealing emerging patterns and development trends.

**Figure 4:**
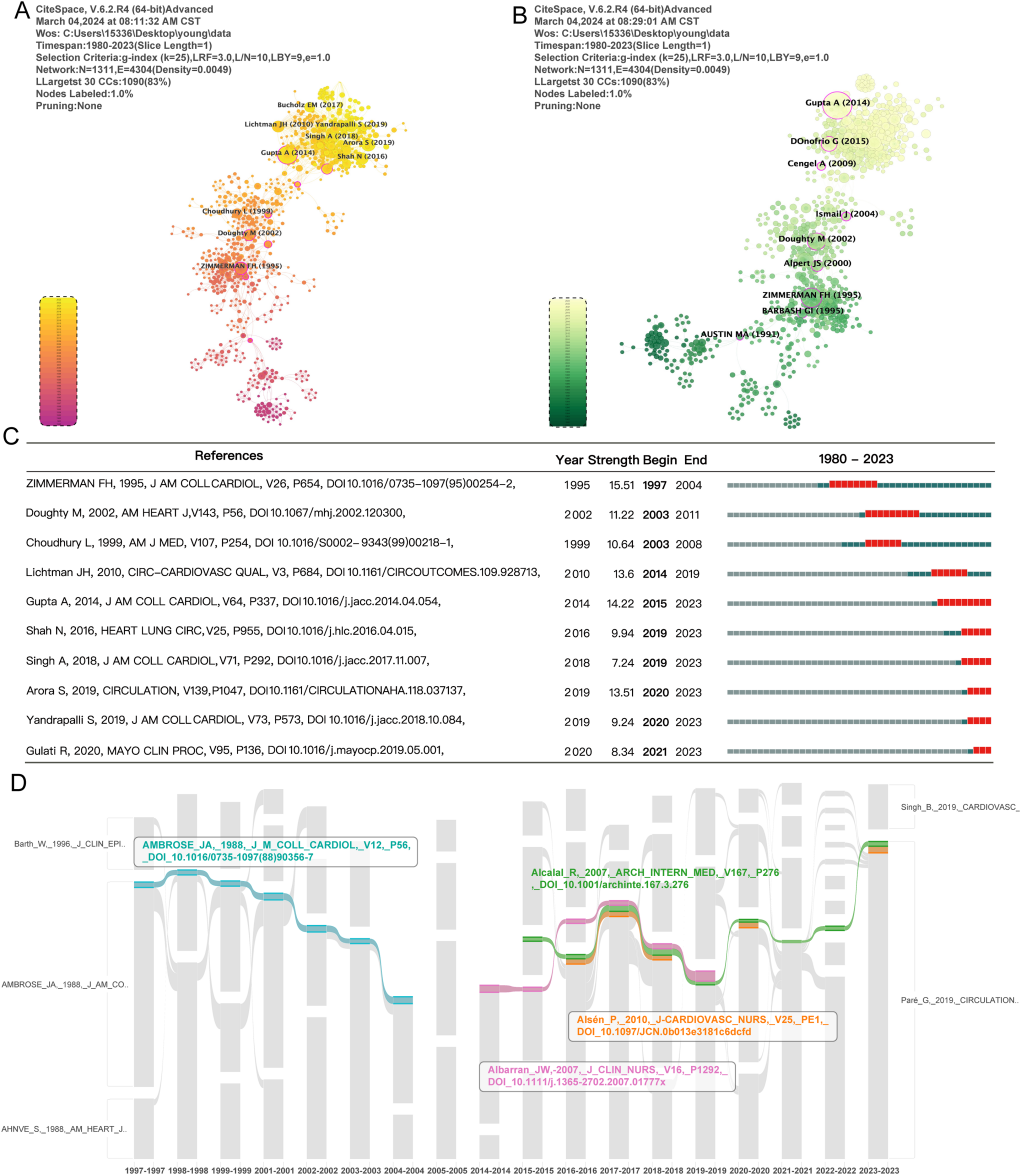
Literature co-citation and clustering visualization analysis. **(A)** The authors of the TOP 10 literature co-citations are listed using CiteSpace. **(B)** Literature centrality is calculated, and the pink circles represent the papers with high centrality of literature centrality. **(C)** Statistics of the TOP 10 articles in terms of outbreak intensity. **(D)** The impact flow chart of literature citation durations by MapEquation visualization and analysis to filter out the key literature that has been continuously cited for more than 5 years.

In the visualization map, which contains links and nodes, nodes with high centrality represent research hotspots or significant turning points in the field. As shown in Figure 4A, we listed the authors with the TOP 10 co-citations of the literature and counted the number of citations, and the detailed data can be seen in Supplementary Table S8. Among them, Gupta A published in J AM Coll Cardiol in 2014, Arora S published in Circulation in 2019, and ZIMMERMAN FH published in published in J AM Coll Cardiol in 1995 were ranked in the TOP 3 with 59, 32, and 28 citations, respectively. In Figure 4B, pink circles represent papers with high centrality. Detailed information on the TOP 10 centrality papers is presented in Supplementary Table S9. Among them, the papers published by Ismail J in 2004 in Heart, Cengel A in 2009 in J Postgrad Med, and ZIMMERMAN FH in 1995 in J AM Coll Cardiol ranked the top three, with mediational centrality of 0.27, 0.24, and 0.19, respectively. These papers on the knowledge network of myocardial infarction in young people play a crucial role as hubs for knowledge dissemination and communication. This helps us identify and predict the key nodes in the knowledge network as well as future trends.

Moreover, Figure 4C shows we counted the outbreak intensity and listed the articles with the TOP 10 outbreak intensity. Among them, ZIMMERMAN FH's document published in J AM Coll Cardiol in 1995 has the highest burst intensity of 15.51, which is at the top of the list. Arora S's article published in Circulation in 2019 has the highest burst intensity of 13.51 between 2020 and 2023. This suggests that scientists are interested in youth myocardial infarction risk factors and trends continue to be of interest, especially regarding gender differences. Exploring the pathological mechanisms associated with myocardial infarction in youth remains a hot research direction.

Additionally, Figure 4D illustrates a flowchart of literature citation durations and filters out key literature consistently cited in the past five years. First, between 1997 and 2004, the Ambrose study evaluated the relationship between cardiovascular lesions and myocardial infarction using coronary angiography. Based on this study, myocardial infarction often results from an underlying cardiovascular lesion. It is difficult to predict the location of subsequent infarcts based on initial coronary angiography (33). Second, John W. Albarran, M.S., studied differences in myocardial infarction symptoms between men and women from 2014 to 2019. He found that gender significantly affects symptom presentation and perception. This underscores the importance of recognizing these differences to improve health behaviors and early diagnosis in women (34). Third, research has been consistently cited for six years. Pia Alsén and Eva Brink found that disease perceptions affect prognosis in myocardial infarction patients, underscoring the need for targeted rehabilitation strategies (35). Finally, in a study on acute myocardial infarction diagnosis, Alcalay et al. predicted the incidence of acute coronary syndromes by evaluating nonspecific troponin. The study continued to receive citations during 2016–2018 and is again in the spotlight in 2023 (36). This study has been consistently cited since 2015. In summary, the above literature has provided an important foundation for the study of myocardial infarction in youth and has contributed to the continued progress of the field.

**Figure 5:**
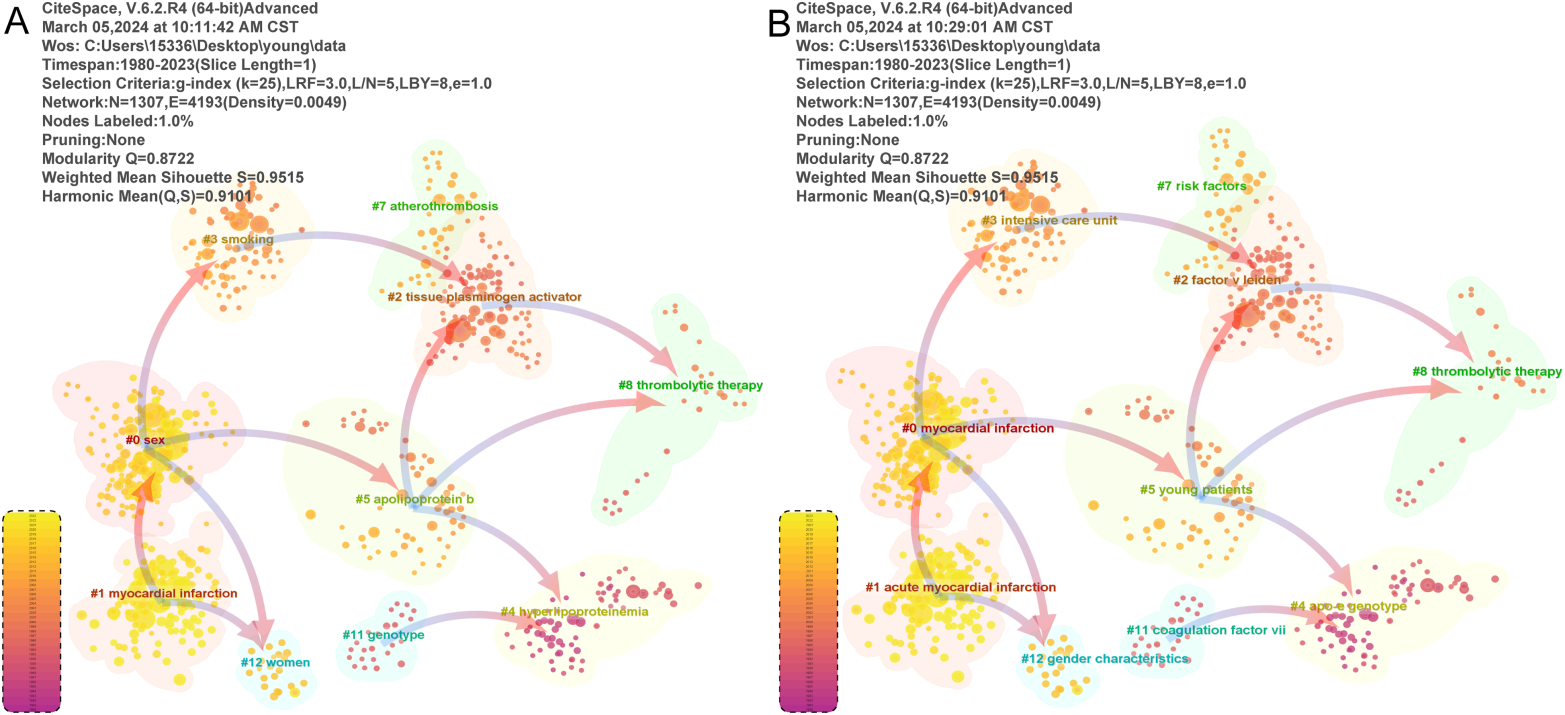
Literature clustering visualization and analysis. **(A,B)** Are labeled with LLR and LSI using the parameter of g-index 25, respectively. The direction of the arrows indicates the source of the literature clusters, i.e., the literature clusters that the arrows are pointing to; the dependencies between the clusters are visualized.

In addition, we visualized and analyzed the literature by clustering using g-index 25, and the results are shown in Figures 5A,B. Ten clusters were obtained, and they were labeled with LLR and LSI, respectively. The direction of the arrows indicates the source of the literature clusters, i.e., the literature clusters pointed at by the arrows. Among them, “#8 Thrombolytic therapy” had identical labels in the LLR and LSI classifications. Specifically, “#0” relates to gender and myocardial infarction; “#1” relates to myocardial infarction and acute myocardial infarction; “#2” relates to tissue plasminogen activator and coagulation factor V mutations; “#3” relates to smoking and intensive care units; “#4” relates to hyperlipoproteinemia and ApoE gene polymorphisms; “#5” relates to apolipoprotein B and young patients; “#7” relates to atherosclerosis and risk factors; “#11” relates to coagulation factor VII and genetic polymorphisms; and “#12” relates to female and gender characteristics. This suggests that gender, smoking, coagulation factors, apolipoproteins, and gene polymorphisms are key research areas in the field of myocardial infarction in youth.

### 3.6 Analysis of journals and co-cited journals

Using CiteSpace's “Overlay Maps” feature, we conducted an in-depth analysis of journals in the field of myocardial infarction in young adults, with a focus on identifying high-impact journals and papers to guide academic research. In Supplementary Figure S7A, cited journals play a key role in providing the necessary knowledge base for referencing literature. In the overlay, the green pathway reveals that studies published in journals related to “Drugs, Medicine, and Clinics” often cite journals in the fields of “Molecular, Biology, and Genetics” and “Nursing, Cardiology, and Pharmacology”. “Nursing, Cardiology and Pharmacology”. Here, the Z-value represents hotness, which is used to assess the relative importance or concentration of a topic or keyword in a literature collection. A higher Z-value indicates a higher level of hotness of the topic in a literature collection. The F-value, on the other hand, refers to the frequency of occurrence of a topic or keyword in a particular literature collection. In bi-period mapping, the F-value is used to compare the change in a topic in a literature collection between two periods. For example, the trend of the F-value of a topic in the first and second periods of the literature collection can reflect whether the research hotspot has changed. Here, the frequency and intensity of citations in the field of “Nursing, Cardiology, and Pharmacology” are high, and these citations are mainly concentrated in the journals Circulation and J AM Coll Cardiol. According to these findings, myocardial infarction in the young and other disciplines are highly interconnected and mutually reinforcing.

Finally, we used the “Bradford's Law” module of the bibliography tool to conduct an in-depth analysis of the most prolific and influential journals in the field of myocardial infarction in young adults. In Supplementary Figure S7B, the overall pattern of journals for young adults with myocardial infarction is shown. Core journals are identified in gray, totaling 22, of which the International Journal of Cardiology, Circulation, and Cureus Journal of Medical Science rank in the top three. In Supplementary Table S10, key information such as Cited score, H-index, Open access (OA), JCR partition, and impact factor of these 20 core journals is detailed. Notably, Circulation stands out with its high Cited Score and H-index, highlighting its notable contribution to the field of myocardial infarction in young adults, with an impact factor of 37.8 and a JCR partition of Q1, as well as not being OA. Similarly, the journal European Heart Journal reached the highest JCR impact factor level with 39.3 points. This was also for the JCR partition of Q1 and with the attribute of not OA. In summary, we identified journals with significant influence in the field of myocardial infarction in youth. These journals provide valuable references for related academic research and submission.

## 4 Discussion

### 4.1 The global status of myocardial infarction research among young people

By statistically analyzing studies in the field of myocardial infarction in young adults between 1980 and 2023, we observed a growing trend in the number of annual publications. However, the annual citation trend went through four phases, including a fluctuating period, a stabilizing period, an explosive growth period, and a slow decline period. We synthesized this phenomenon and suggested that possible underlying factors include declining study quality, fragmentation of research areas, citation lag, citation habits, and changes in standards.

In recent years, significant progress has been made globally in the study of myocardial infarction in young people. This focuses on an in-depth exploration of the mechanisms and risk factors associated with myocardial infarction in young people, improvement of diagnosis and treatment, and improvement of prognosis.

### 4.2 Regarding the mechanisms of myocardial infarction in youth

Several studies have pointed out that myocardial infarction is closely related to inflammation and metabolic changes. In this regard, short chain fatty acids and oxidized trimethylamine are significant regulators of inflammation (37). Secondly, inflammatory factors such as C-reactive proteins, interleukins 1 and 6 also play an important role in this process (38). In addition, oxidative stress has been the focus of academic research in this field, such as in-depth studies on mitochondrial dysfunction (39). In particular, in recent years, scholars have shown great interest in exploring the direction of cell death and repair, including emerging research directions such as apoptosis, iron-death, and copper-death (40, 41), and they have been actively explored at the level of gene polymorphisms and molecular pathways. The coagulation system has also received much attention, involving fibrinogen, fibrinogen activators and coagulation factors (42). In addition, the study of lipids, such as apolipoprotein B, apolipoprotein A1, and apolipoprotein F, is also a hot topic in current research.

### 4.3 Risk factors for myocardial infarction in youth

The risk factors for myocardial infarction combined with obstructive coronary artery disease in young adults are similar to those for myocardial infarction in middle-aged and older adults, including family history, smoking, atherosclerosis, hypertension, insulin resistance, and hyperlipidemia. Familial dyslipidemia also increases myocardial infarction risk in youth. The etiology of myocardial infarction combined with nonobstructive coronary myocardial infarction (MINOCA) in young people includes endocardial coronary artery spasms, coronary embolization, and thrombosis in addition to atherosclerosis. In addition, spontaneous coronary artery dissection (SCAD) has been implicated as a cause of myocardial infarction episodes. SCAD is common in female patients, with onset occurring between the ages of 23–44 years, and is particularly prevalent within the first week after childbirth (43). Academic literature confirms a significant association of depression and anxiety states with the development of myocardial infarction in youths, and even marital stress has been found to influence the incidence of myocardial infarction in youths (44). Substance abuse, such as cocaine, marijuana, and methamphetamine, increases myocardial infarction in youth (45). In addition to atherosclerosis, spontaneous stenosis of the coronary arteries as well as disorders such as coronary artery spasms have been implicated in myocardial infarction episodes (46).

### 4.4 Diagnostic and therapeutic options

In terms of serum markers, creatine phosphokinase isoenzyme CK-MB, troponin I (TnI/T) and high-sensitivity C-reactive protein are useful for early myocardial infarction diagnosis. A study of young Indian patients with myocardial infarction found that heart-type fatty acid binding protein (H-FABP) may be an effective cardiac marker for early diagnosis in young patients with myocardial infarction (47). Coronary angiography remains the key test for the diagnosis of coronary vasculopathy, and emergency percutaneous vascular intervention (PCI) is the treatment of choice for patients with ST-segment elevation myocardial infarction (48). With the continuous development and application of new devices such as bioabsorbable stents and pharmacological balloons, the PCI technique has been optimized. While coronary artery bypass grafting (CABG) may be more appropriate for patients who are not candidates for PCI and are at risk of extensive myocardial ischemia. PCI may be a more prioritized treatment strategy in patients with non-ST elevation myocardial infarction with single or double coronary artery disease, while CABG may be more appropriate in the presence of complex multibranch disease, especially with left anterior descending branch or left main trunk involvement.

Furthermore, cardiac magnetic resonance and intravascular imaging tests are critical in diagnosing and treating MINOCA patients. A definitive diagnosis of SCAD usually requires coronary angiography supplemented by intravascular ultrasound and optical correlation tomography (49). Treatment is mainly based on antiplatelet aggregation, lipid-lowering to stabilize plaque, inhibition of myocardial remodeling, and heart rate reduction. However, in recent years, academia has shown particular interest in novel lipid-lowering agents, focusing on targeted therapies at the protein, mRNA, and gene levels.

### 4.5 Strength and limitations

The study has the following strengths:

(1) This study is the first paper to systematically and comprehensively survey and summarize global trends and frontiers in the field of myocardial infarction in young adults using bibliometric analysis.
(2) This study, based on the WoSCC database and covering the period 1980–2023, aims to provide a comprehensive assessment of the history and research hotspots of myocardial infarction in adolescents. It also provides insight into the current state of basic and clinical research on myocardial infarction in young people.

The study has the following limitations:

(1) Although the WoSCC database is widely recognized as a database of relative excellence, the fact that only that database was used in this paper may result in a less comprehensive data collection.
(2) Given that papers lag, this study set the cutoff for December 2023 when screening papers. This may lead to incomplete data.

## 5 Conclusion

This study used bibliometric methods to comprehensively explore myocardial infarction in young adults. Firstly, it statistically analyzed publication and citation trends, revealing annual publication growth trends and four phases of citation trends: fluctuating, stabilizing, exploding, and declining. Secondly, it presented an in-depth understanding of authors, countries, institutions, and collaborations, highlighting the main contributors and potential collaborations. Lastly, it analyzed keywords, co-cited papers, and high-impact journals to identify research hotspots and trends to assist researchers in contributing to the field.

## Data Availability

The original contributions presented in the study are included in the article/Supplementary Material, further inquiries can be directed to the corresponding author.
