# Supplementary material for: Bibliometric analysis of youth myocardial infarction research (1980–2023)
Source: Front Cardiovasc Med. 2024 Nov 26;11:1478158. doi: 10.3389/fcvm.2024.1478158 (PMC11628501; doi:10.3389/fcvm.2024.1478158)
Supplement: Supplementary file 1 [file Datasheet1.pdf]

Supplementary Materials

Figure. S1. Graph of annual publication trends and citation trends. 2
Figure. S2. National and institutional publication contributions. 3
Figure. S3. TOP10 burst strength author. 4
Figure. S4: Keyword visualization analysis using CiteSpace software and LLR clustering analysis with clustering parameter set to TOP N=40, 22 clusters were obtained. 5
Figure. S5: Keyword visualization and analysis using CiteSpace software with LLR clustering analysis, setting the clustering parameter as TOP N=50, 22 clusters were obtained. 6
Figure. S6: Keyword fuzzy set theme analysis. 7
Figure.S7: Journal visualization analysis. 8
Table S1. Top 10 authors according to H-index, including counts, G/M index and total citations.... 9
TableS2: Number of articles published by the top 10 authors. 10
TableS3: G-index of the top 10 authors. 11
TableS4: M-index of the top 10 authors. 12
Table S5. Keyword LLR clustering parameters Top N=30, #0-#23Cluster Size, Silhouette, Average Year and labels 13
Table S6: Keyword LLR clustering parameters TOP N=40,#0-#22Cluster Size, Silhouette, Average Year and labels. 15
Table S7: Keyword LLR clustering parameters TOP N=50, #0-#22Cluster Size, Silhouette, Average Year and labels. 16
Table S8: The 10 most co-cited documents are listed below, along with citation counts, journals, and DOI. 17
Table S9: Top 10 papers and DOI details for literature mediated centrality. 18
Table S10: Top 20 journals: cited score, H-index, JCR impact factor, quartiles and OA details. ... 19

Figure. S1. Graph of annual publication trends and citation trends.

(A) Annual publication trends from 1980-2023 show an overall growth trend; the blue area represents the slow growth phase from 1980-2020, the red area represents the stable growth phase (2000-2020); the green area is the explosive growth phase from 2020 to 2023. (B) Trends in annual citation frequency, roughly divided into the first stage (1980-1998) is a period of fluctuation; the second stage (1998-2008) is a period of stabilization; the third stage (2008-2017) is a period of explosive growth; and the fourth stage (2017-2023) is a period of slow decline.

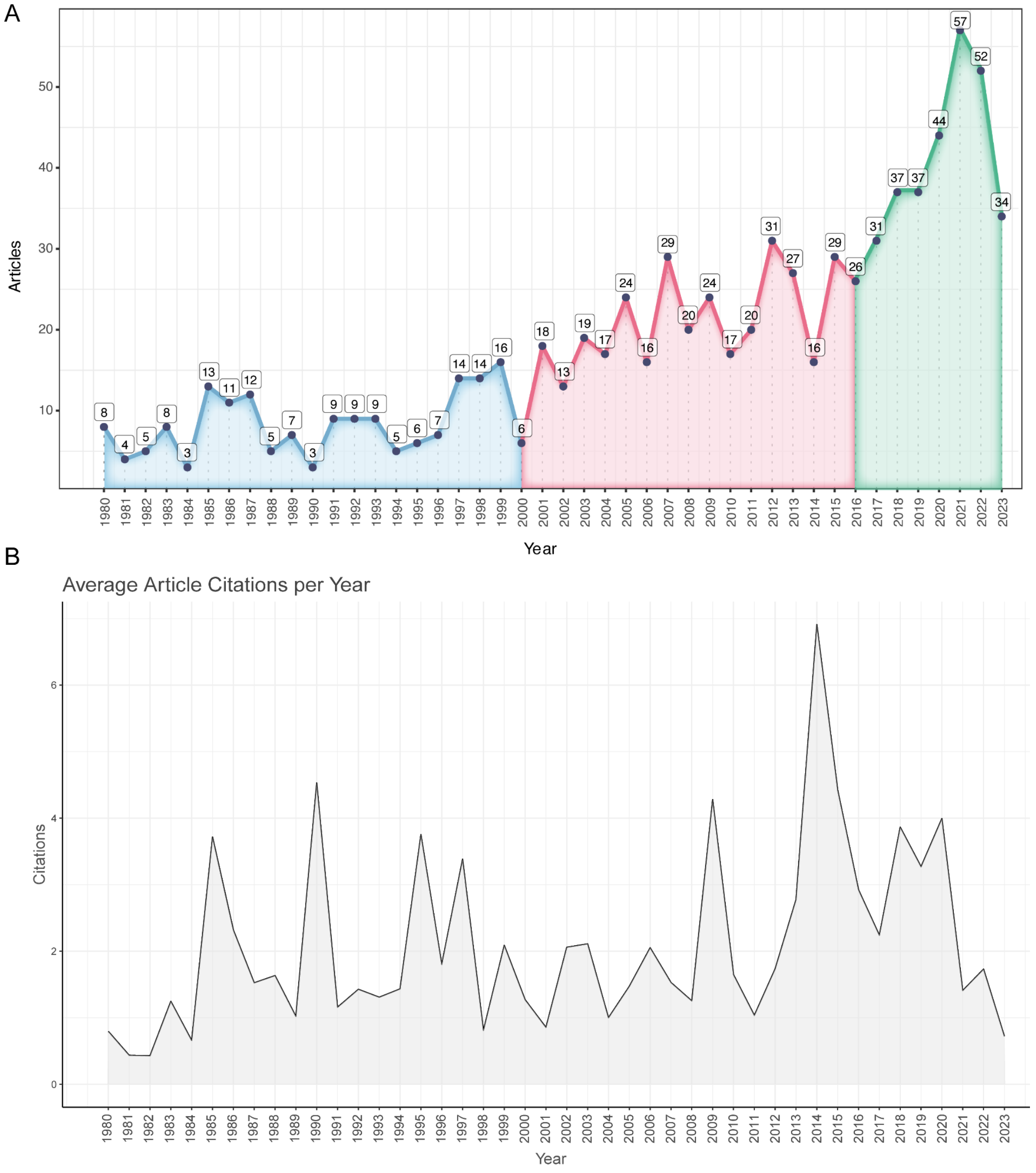

Figure. S2. National and institutional publication contributions.

(A) Cooperation between countries is categorized in the figure in green, blue and orange. The size of the dots reflects the number of publications in each country, while the connecting lines reflect the intensity of the cooperation. (B) The graph categorizes inter-agency cooperation into, blue and green. The size of the dots indicates the number of publications, while the width of the connecting lines indicates the intensity of cooperation.

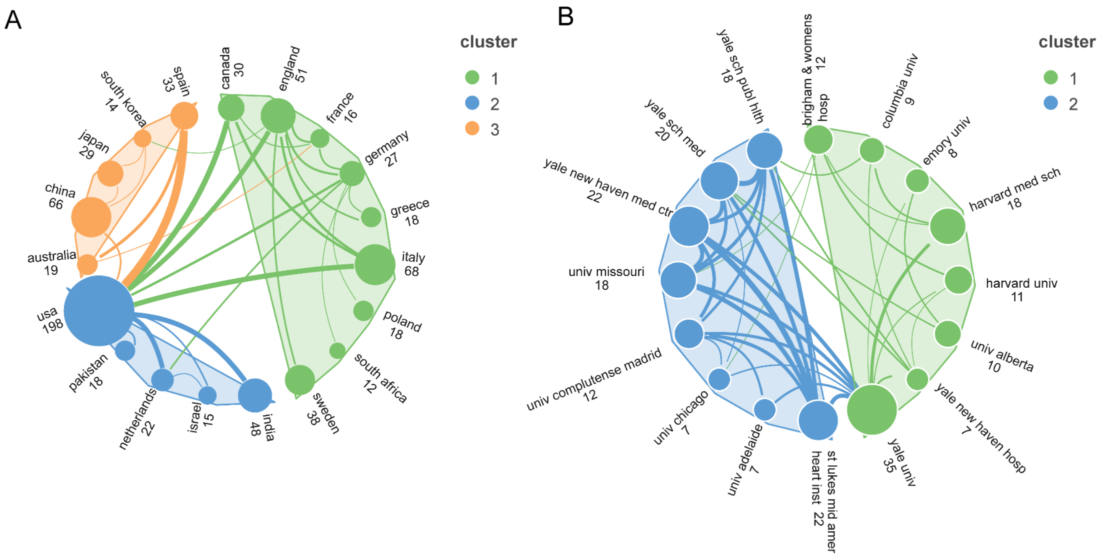

Figure. S3. TOP10 burst strength author.

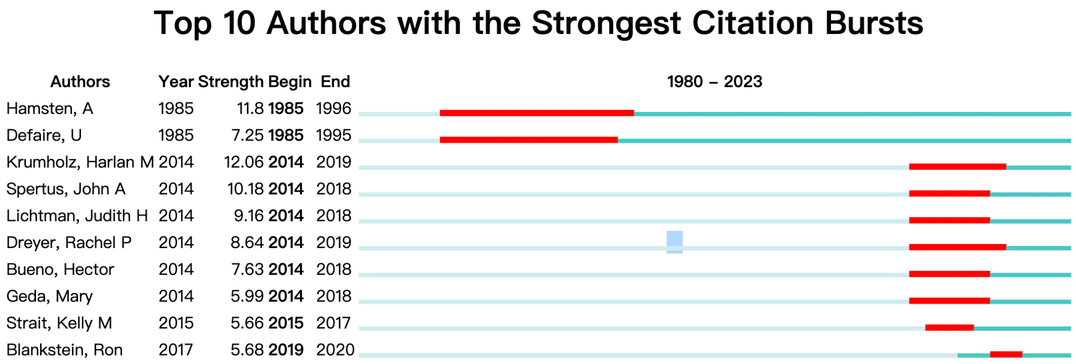

Figure. S4: Keyword visualization analysis using CiteSpace software and LLR clustering analysis with clustering parameter set to TOP N=40, 22 clusters were obtained.

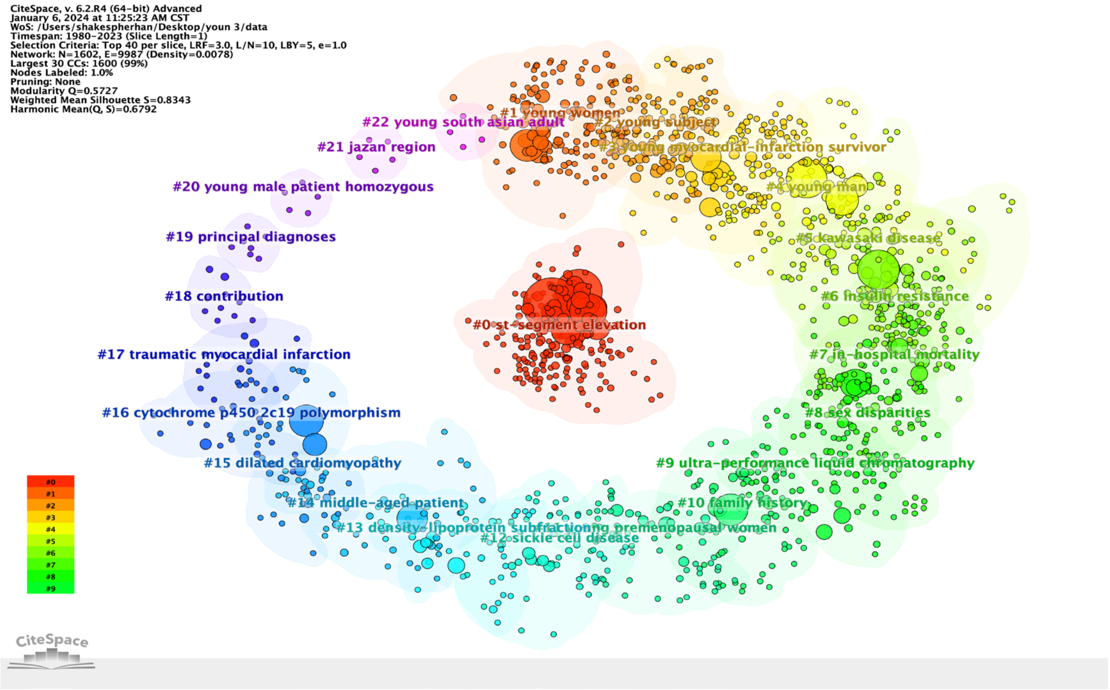

Figure. S5: Keyword visualization and analysis using CiteSpace software with LLR clustering analysis, setting the clustering parameter as TOP N=50, 22 clusters were obtained.

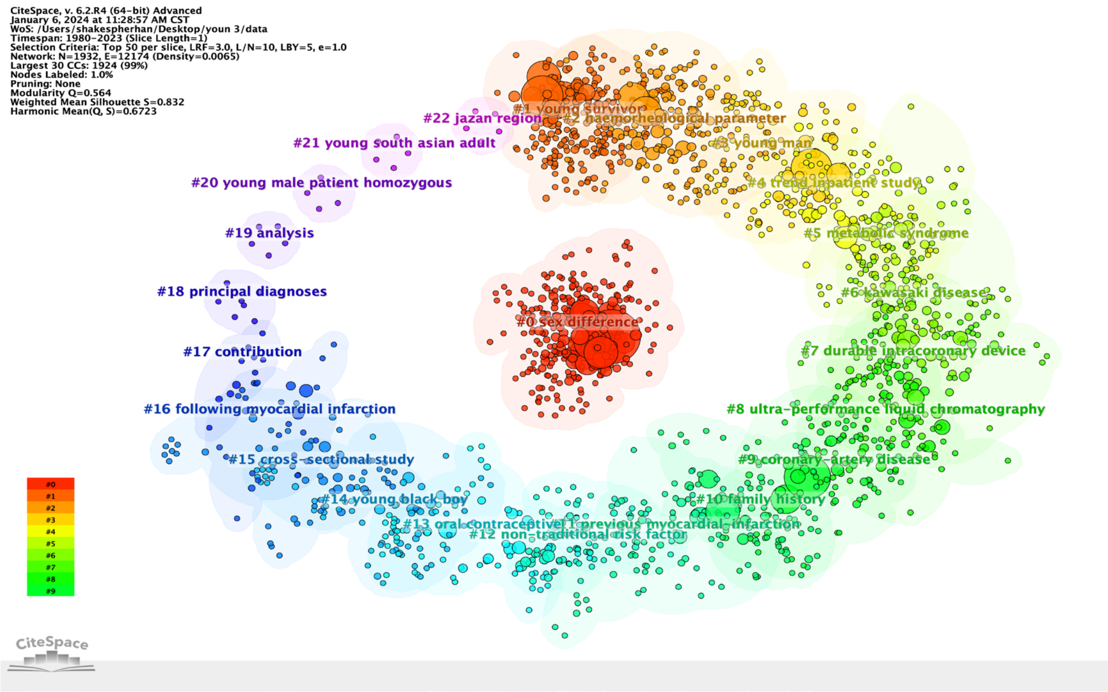

Figure. S6: Keyword fuzzy set theme analysis.

The keywords are divided into 4 quadrants, where the horizontal coordinate represents centrality and the vertical coordinate represents density. The upper right quadrant presents "Motor Themes keywords"; the upper left quadrant represents "Niche Themes keywords"; the lower right quadrant represents "Basic Themes keywords"; the lower left quadrant represents "Emerging Themes keywords"; and the lower left quadrant represents "Emerging Themes keywords". The lower right limit represents "Basic Themes keywords"; the lower left limit is "Emerging or Declining Themes keywords".

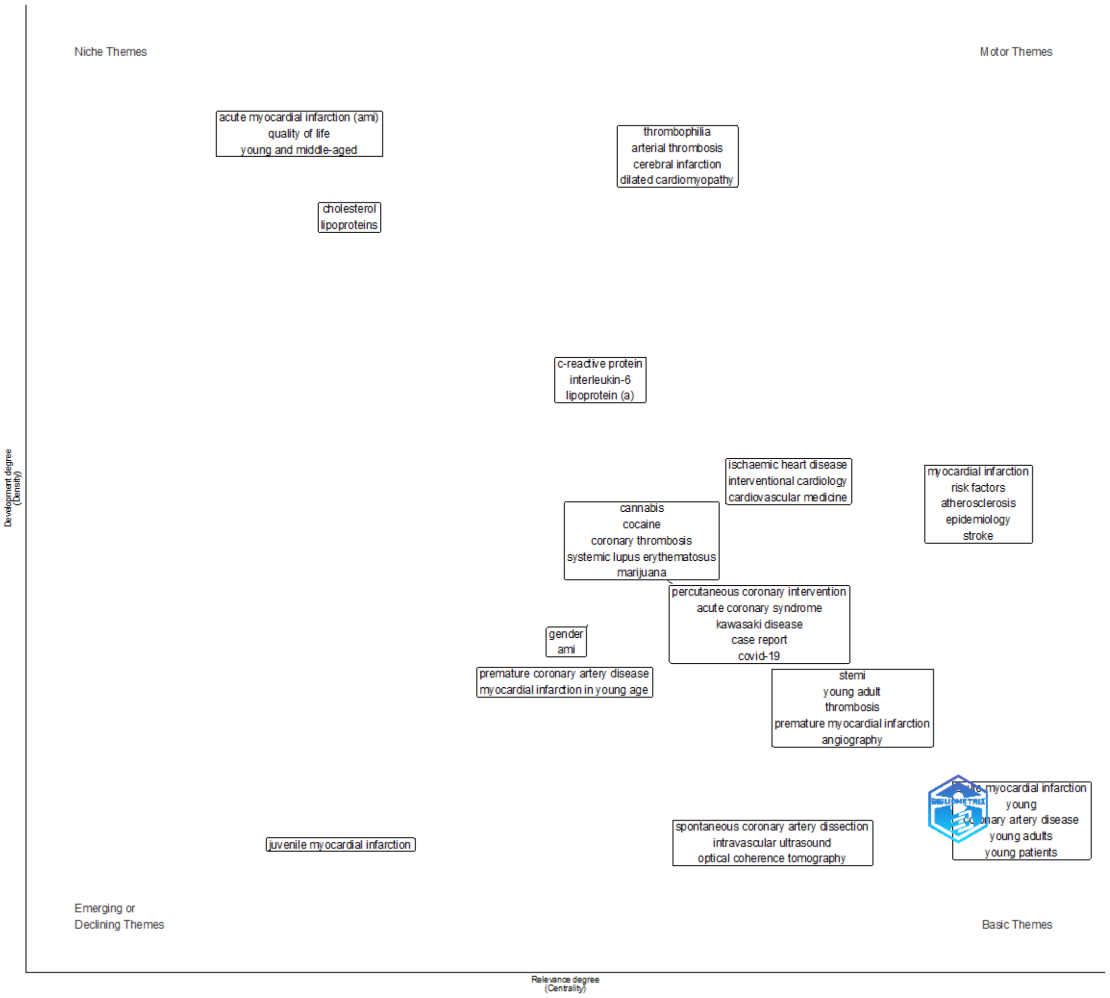

Figure.S7: Journal visualization analysis.

(A) Dual mapping overlay showing journals. circulation and J am Coll Cardiol are the major cited journals. (B) By using the "Bradford's Law" module in the bibloshiny tool, the top 20 influential journals are filtered.

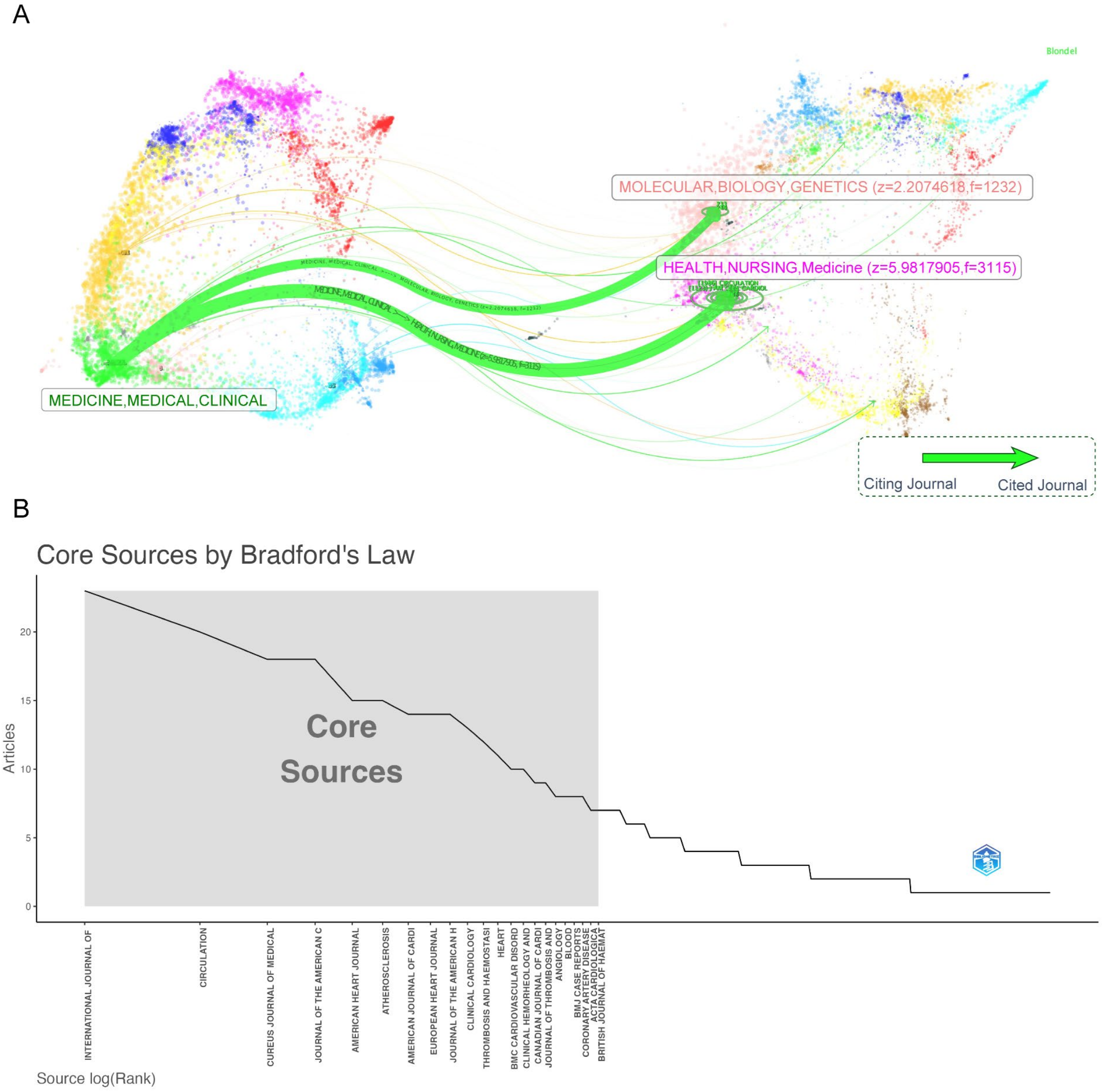

Table S1. Top 10 authors according to H-index, including counts, G/M index and total citations

Ranks	Authors	h_index	g_index	m_index	TC	NP	PY_start
1	KRUMHOLZ HM	22	34	1.222	2097	34	2006
2	HAMSTEN A	20	27	0.513	3597	27	1985
3	SPERTUS JA	19	27	1.056	1861	27	2006
4	D'ONOFRIO G	18	29	1.8	1678	29	2014
5	LICHTMAN JH	18	22	1	1824	22	2006
6	ROSENDAAL FR	16	19	0.593	1684	19	1997
7	BUENO H	14	18	1.4	961	18	2014
8	DREYER RP	14	26	1.4	987	26	2014
9	BHATT DL	12	14	1.333	890	14	2015
10	BLANKSTEIN R	12	15	1.714	736	15	2017
11	GEDA M	12	14	1.2	1235	14	2014

TableS2: Number of articles published by the top 10 authors.

Rank	Authors	Counts
1	Krumholz,Harlan M	33
2	D'onfrio,Gail	29
3	Hamsten,A	27
4	Spertus John A	27
5	Dreyer,Rachel P	26
6	Lichtman,Judith H	22
7	Rosendaal, FR	19
8	Bueno,Hector	18
9	Blankstein,Ron	15
10	Spatz,Erica S	15
11	Bhatt, Deepak L	14
12	Defaire,U	14
13	Geda,Mary	14
14	Nasir, Khurram	14

TableS3: G-index of the top 10 authors.

Rank	Authors	G_index
1	Krumholz,Harlan M	34
2	D'onfrio,Gail	29
3	Hamsten,A	27
4	Spertus John A	27
5	Dreyer,Rachel P	26
6	Lichtman,Judith H	22
7	Rosendaal, FR	19
8	Bueno,Hector	18
9	Blankstein,Ron	15
10	Spatz,Erica S	15
11	Bhatt, Deepak L	14
12	Geda,Mary	14
13	Nasir, Khurram	14
14	Singh A	13

TableS4: M-index of the top 10 authors.

Rank	Authors	M_index
1	D'onfrio,Gail	1.8
2	Biery DW	1.75
3	Blankstein,Ron	1.714
4	Nasir, Khurram	1.571
5	Berman AN	1.5
6	Di Carli MF	1.429
7	Singh A	1.429
8	Bueno,Hector	1.4
9	Dreyer,Rachel P	1.4
10	Bhatt, Deepak L	1.333
11	Defilippis EM	1.333
12	Qamar A	1.286
13	Krumholz,Harlan M	1.222

Table S5. Keyword LLR clustering parameters Top N=30, #0-#23Cluster Size, Silhouette, Average Year and labels

Cluster ID	Size	Silhouette	Label (LLR)	Average Year
0	201	0.634	acute myocardial infarction (436.02, 1.0E-4)	2005
1	128	0.8	young women (650.44, 1.0E-4)	2003
2	110	0.816	young myocardial-infarction survivor (288.29, 1.0E-4)	2000
3	91	0.838	insulin resistance (285.53, 1.0E-4)	2004
4	76	0.89	kawasaki disease (272.5, 1.0E-4)	2003
5	76	0.842	haemorheological parameter (180.01, 1.0E-4)	2005
6	66	0.907	sickle cell disease (102.16, 1.0E-4)	2001
7	65	0.922	hemostatic disorder (107.64, 1.0E-4)	2003
8	55	0.909	family history (270.83, 1.0E-4)	2003
9	52	0.876	coronary artery (92.51, 1.0E-4)	2004
10	48	0.944	different lipid risk factor (97.13, 1.0E-4)	2005
11	41	0.918	m-mode echocardiography (81.56, 1.0E-4)	1999
12	41	0.905	density-lipoprotein subfraction (77.76, 1.0E-4)	1999
13	37	0.907	thrombus management (75.95, 1.0E-4)	2005
14	27	0.977	cytochrome p450 2c19 polymorphism (44.38, 1.0E-4)	2007

15	21	0.971	young black boy (47.6, 1.0E-4)	1997
16	20	0.959	age (31.86, 1.0E-4)	2003
17	12	0.998	traumatic myocardial infarction (18.85, 1.0E-4)	2009
18	10	1	contribution (15.57, 1.0E-4)	2011
19	9	0.991	highests rate (17.32, 1.0E-4)	2010
20	8	1	myocardial infarction-an exploratory study (18.85, 1.0E-4)	2009
21	8	0.994	comparison (17.94, 1.0E-4)	2003
22	5	1	young male patient homozygous (18.34, 1.0E-4)	2010
23	5	1	young south asian adult (15.71, 1.0E-4)	2004

Table S6: Keyword LLR clustering parameters TOP N=40,#0-#22Cluster Size, Silhouette, Average Year and labels.

ClusterID	Size	Silhouette	Label (LLR)	Average Year
0	219	0.671	st-segment elevation (736.79, 1.0E-4)	2009
1	136	0.774	young women (559.12, 1.0E-4)	2004
2	130	0.803	young subject (186.39, 1.0E-4)	2007
3	113	0.881	young myocardial-infarction survivor (221.5, 1.0E-4)	2002
4	101	0.794	young man (214.07, 1.0E-4)	2007
5	100	0.89	kawasaki disease (306.8, 1.0E-4)	2006
6	97	0.843	insulin resistance (255.91, 1.0E-4)	2004
7	88	0.856	in-hospital mortality (114.53, 1.0E-4)	2003
8	83	0.864	sex disparities (136.58, 1.0E-4)	2010
9	75	0.922	ultra-performance liquid chromatography (124.4, 1.0E-4)	2006
10	70	0.899	family history (222.66, 1.0E-4)	2005
11	65	0.908	young premenopausal women (111.85, 1.0E-4)	2007
12	61	0.885	sickle cell disease (68.47, 1.0E-4)	2004
13	52	0.894	density-lipoprotein subfraction (93.3, 1.0E-4)	2001
14	47	0.928	middle-aged patient (131.16, 1.0E-4)	2010
15	39	0.92	dilated cardiomyopathy (231.74, 1.0E-4)	2004
16	30	0.97	cytochrome p450 2c19 polymorphism (44.18, 1.0E-4)	2007
17	13	0.969	traumatic myocardial infarction (32.97, 1.0E-4)	2009
18	10	0.996	contribution (18.27, 1.0E-4)	2011
19	7	0.996	principal diagnoses (18.27, 1.0E-4)	2015
20	5	1	young male patient homozygous (18.27, 1.0E-4)	2010
21	5	1	jazan region (18.27, 1.0E-4)	2023
22	5	1	young south asian adult (16.69, 1.0E-4)	2004

Table S7: Keyword LLR clustering parameters TOP N=50, #0-#22Cluster Size,

Silhouette, Average Year and labels.

lusterID	Size	Silhouette	Label (LLR)	Average Year
0	294	0.658	sex difference (593.46, 1.0E-4)	2010
1	229	0.742	young survivor (564.86, 1.0E-4)	2004
2	174	0.806	haemorheological parameter (242.81, 1.0E-4)	2007
3	97	0.913	young man (280.86, 1.0E-4)	2006
4	97	0.893	trend inpatient study (187.63, 1.0E-4)	2011
5	97	0.863	metabolic syndrome (168.17, 1.0E-4)	2008
6	92	0.886	kawasaki disease (350.42, 1.0E-4)	2008
7	89	0.898	durable intracoronary device (142.81, 1.0E-4)	2013
8	88	0.894	ultra-performance liquid chromatography (142.6, 1.0E-4)	2007
9	87	0.879	coronary-artery disease (243.75, 1.0E-4)	2006
10	86	0.903	family history (269.24, 1.0E-4)	2006
11	83	0.911	previous myocardial-infarction (135.32, 1.0E-4)	2003
12	81	0.907	non-traditional risk factor (130.08, 1.0E-4)	2009
13	81	0.882	oral contraceptive (205.45, 1.0E-4)	2012
14	74	0.896	young black boy (102.79, 1.0E-4)	2011
15	58	0.895	cross-sectional study (191.26, 1.0E-4)	2011
16	30	0.948	following myocardial infarction (75.95, 1.0E-4)	2014
17	11	0.994	contribution (33.88, 1.0E-4)	2012
18	7	0.997	principal diagnoses (18.73, 1.0E-4)	2015
19	5	1	analysis (18.32, 1.0E-4)	2017
20	5	1	young male patient homozygous (18.73, 1.0E-4)	2010
21	5	1	young south asian adult (19.91, 1.0E-4)	2004
22	5	1	jazan region (18.73, 1.0E-4)	2023

Table S8: The 10 most co-cited documents are listed below, along with citation counts, journals, and DOI.

Citation Counts	References	DOI
59	Gupta A, 2014, J AM COLL CARDIOL, V64, P337	10.1016/j.jacc.2014.04.054
32	Arora S, 2019, CIRCULATION, V139, P1047	10.1161/CIRCULATIONAHA.118.037137
28	ZIMMERMAN FH, 1995, J AM COLL CARDIOL, V26, P654	10.1016/0735-1097(95)00254-2
27	Lichtman JH, 2010, CIRC-CARDIOVASC QUAL, V3, P684	10.1161/CIRCOUTCOMES.109.928713
26	Shah N, 2016, HEART LUNG CIRC, V25, P955	10.1016/j.hlc.2016.04.015
22	Doughty M, 2002, AM HEART J, V143, P56	10.1067/mhj.2002.120300
22	Yandrapalli S, 2019, J AM COLL CARDIOL, V73, P573	10.1016/j.jacc.2018.10.084
19	Singh A, 2018, J AM COLL CARDIOL, V71, P292	10.1016/j.jacc.2017.11.007
19	Bucholz EM, 2017, EUR HEART J-ACUTE CA, V6, P610	10.1177/2048872616661847
19	Choudhury L, 1999, AM J MED, V107, P254	10.1016/S0002-9343(99)00218-1

Table S9: Top 10 papers and DOI details for literature mediated centrality.

Sigma	References	DOI
14.85	ZIMMERMAN FH, 1995, J AM COLL CARDIOL, V26, P654	10.1016/0735-1097(95)00254-2
8.27	Gupta A, 2014, J AM COLL CARDIOL, V64, P337	10.1016/j.jacc.2014.04.054
3.19	Doughty M, 2002, AM HEART J, V143, P56	10.1067/mhj.2002.120300
2.20	Alpert JS, 2000, J AM COLL CARDIOL, V36, P959	10.1016/S0735-1097(00)00804-4
2.13	DOnofrio G, 2015, CIRCULATION, V131, P1324	10.1161/CIRCULATIONAHA.114.012293
1.84	BARBASH GI, 1995, EUR HEART J, V16, P313	
1.63	HAMSTEN A, 1986, CIRCULATION, V73, P1097	10.1161/01.CIR.73.6.1097
1.60	Lichtman JH, 2010, CIRC-CARDIOVASC QUAL, V3, P684	10.1161/CIRCOUTCOMES.109.928713
1.39	Canto JG, 2012, JAMA-J AM MED ASSOC, V307, P813	10.1001/jama.2012.199
1.38	Egred M, 2005, POSTGRAD MED J, V81, P741	10.1136/pgmj.2004.027532

Table S10: Top 20 journals: cited score, H-index, JCR impact factor, quartiles and OA details.

Rank	Journal	Cited score	H-index	IF (2023)	Quatile	OA
1	INTERNATIONAL JOURNAL OF CARDIOLOGY	7.0	108	3.2	Q2	No
2	CIRCULATION	42.1	570	35.5	Q1	No
3	CUREUS JOURNAL OF MEDICAL SCIENCE	-	-	1	-	No
4	JOURNAL OF THE AMERICAN COLLEGE OF CARDIOLOGY	40.8	394	21.7	Q1	No
5	AMERICAN HEART JOURNAL	8.5	173	3.7	Q2	No
6	ATHEROSCLEROSIS	9.5	159	4.9	Q1	No
7	AMERICAN JOURNAL OF CARDIOLOGY	4.9	206	2.3	Q3	No
8	EUROPEAN HEART JOURNAL	29.2	265	37.6	Q1	No
9	JOURNAL OF THE AMERICAN HEART ASSOCIATION	8.6	60	5	Q2	Yes
10	CLINICAL CARDIOLOGY	4.8	67	2.4	Q3	No
11	THROMBOSIS AND HAEMOSTASIS	10.5	178	5	Q1	No
12	HEART	10.4	165	5.1	Q1	No
13	BMC CARDIOVASCULAR DISORDERS	3.0	48	2	Q4	Yes
14	CLINICAL HEMORHEOLOGY AND MICROCIRCULATION	4.2	48	2.1	Q3	No
15	CANADIAN JOURNAL OF CARDIOLOGY	7.5	77	5.8	Q2	No
16	JOURNAL OF THROMBOSIS AND HAEMOSTASIS	22.1	154	5.5	Q1	No
17	ANGIOLOGY	5.4	59	2.6	Q3	No
18	BLOOD	21.9	426	21	Q1	No
19	MJ CASE REPORTS	1.2	-	-	-	Yes

20	CORONARY ARTERY DISEASE	2.4	58	1.5	Q4	No
